# Batch Preparation and Performance Study of Boehmite-Based Electrospun Nanofiber Separators for Lithium-Ion Batteries

**DOI:** 10.3390/molecules29163938

**Published:** 2024-08-21

**Authors:** Wenfei Ding, Yuxing Liu, Lan Xu

**Affiliations:** 1 National Engineering Laboratory for Modern Silk, College of Textile and Engineering, Soochow University, 199 Ren-Ai Road, Suzhou 215123, China; 20225215012@stu.suda.edu.cn (W.D.); 20245215008@stu.suda.edu.cn (Y.L.); 2 Jiangsu Engineering Research Center of Textile Dyeing and Printing for Energy Conservation, Discharge Reduction and Cleaner Production (ERC), Soochow University, Suzhou 215123, China

**Keywords:** lithium-ion batteries, separators, electrospinning, nanofibers, batch preparation

## Abstract

The design and preparation of high-performance separators for lithium-ion batteries (LIBs) have far-reaching practical significance in enhancing the overall performance of LIBs. Electrospun nanofiber separators (ENSs) have the characteristics of large specific surface area, high porosity, small pore size and good affinity with the electrolyte, making them become ideal candidates for LIB separators. In this work, polyacrylonitrile (PAN)/polyurethane (PU) (PAU) ENSs loaded with boehmite (BM) particles (BM/PAU ENSs) were mass-produced using spherical section free surface electrospinning (SSFSE), and used as LIB separators. Their morphology, structures and performances were tested and characterized. The results showed that all BM/PAU ENSs maintained excellent thermal dimensional stability in the range of 140–180 °C, and had good electrolyte wettability and high porosity. The composite BM/PAU-2 ENS with the best performance had a porosity of 52.5%, an electrolyte uptake rate of 822.1%, and an ionic conductivity of 1.97 mS/cm. Additionally, the battery assembled with BM/PAU-2 separator also demonstrated best electrochemical performance, cycling performance, and rate capability, with a capacity retention rate of 94.4% after 80 cycles at 0.5 C, making it a promising high-performance separator for LIBs.

## 1. Introduction

Rechargeable batteries can effectively convert chemical energy into electrical energy and rely on electrochemical reactions to store energy. Compared with other rechargeable batteries, lithium-ion batteries (LIBs), as a kind of energy storage device with excellent performance, have occupied a dominant position in the field of chemical energy storage due to their slow self-discharge rate, long service life, relatively high energy and power density, and no memory effect. These advantages fully satisfy the urgent requirements for lightweight and miniaturization of power supplies in practical applications, making LIBs widely used in various fields from portable electronic devices to electric vehicles [1,2].

As an important support for the internal structure of LIBs, the separator, although not directly involved in the electrochemical reaction process of the battery, plays a crucial role in hindering electron transfer, isolating the positive and negative electrodes, providing ion transport channels, as well as improving the stability and safe operation of the battery. Currently, polyethylene (PE) and polypropylene (PP) separators with micrometer-sized pore sizes have been commercialized and widely used in LIBs due to their low cost and good mechanical properties [3]. However, PE and PP have the disadvantages of low porosity and poor electrolyte wettability and heat resistance, making it difficult for them to meet the energy storage requirements for LIBs, including their high safety and high energy density [4]. Electrospun nanofiber separators (ENSs) with a three-dimensional network structure are formed by stretching a fine stream of polymers, which contain a large number of pores, providing more channels for lithium-ion transport [5].

Single-needle electrospinning (SNE) is the simplest among various electrospinning methods, but its low production efficiency limits the industrial application of ENSs. On this basis, the proposed multi-needle electrospinning improves the efficiency of electrospinning [6], but the electric fields formed by the multi-jets interfere with each other, and there are still needle blockage problems. Needleless electrospinning (NES) solves these problems by allowing the spinning solution to directly overcome the surface tension of the free liquid surface to form a large number of jets, which are stretched into nanofibers and collected on the receiving device [7]. Various types of NES have been proposed for batch preparation of nanofibers, such as rotating electrospinning [8], bubble electrospinning [9], free surface electrospinning (FSE) [10], and so on. In our previous work [11], the optimal polyacrylonitrile (PAN)/polyurethane (PU) (PAU) ENSs (PAN:PU = 8:2) were prepared in batch by spherical section FSE (SSFSE), with a yield (5.4 g/h) much higher than SNE, exhibiting good mechanical properties and excellent thermal stability, making it an ideal base for the improvement of ENSs. Boehmite (BM) is a preferred inorganic composite material due to its excellent thermal stability and flame retardancy, as well as good chemical and electrochemical stability [12,13].

In this paper, BM/PAU ENSs with different contents of BM particles were efficiently prepared by using SSFSE. The effects of BM content on the morphology, structure, and performance of the composite ENSs were investigated, and the optimal BM/PAU ENS was determined. Ultimately, the LIB assembled with the optimal ENS exhibited better performance in terms of impedance, cycling, and rate.

## 2. Results

### 2.1. Properties of Spinning Solutions

The viscosity and conductivity of spinning solutions with different BM contents are shown in Table 1. It could be found that with the rise of BM content, the viscosity and conductivity of solutions showed a gradual increasing trend, which may be due to the fact that as the content of BM particles increased, the spacing between particles decreased, which increased the interaction force between them. At the same time, the friction between polymer molecular chains also increased, which limited the movement of polymer molecular chains and significantly accelerated the viscosity of the system, making the solution more viscous. However, high solution viscosity can cause nanofiber adhesion during the spinning process, affecting the uniformity of nanofiber distribution.

### 2.2. Morphology and Structure of ENSs

**Figure 1:**
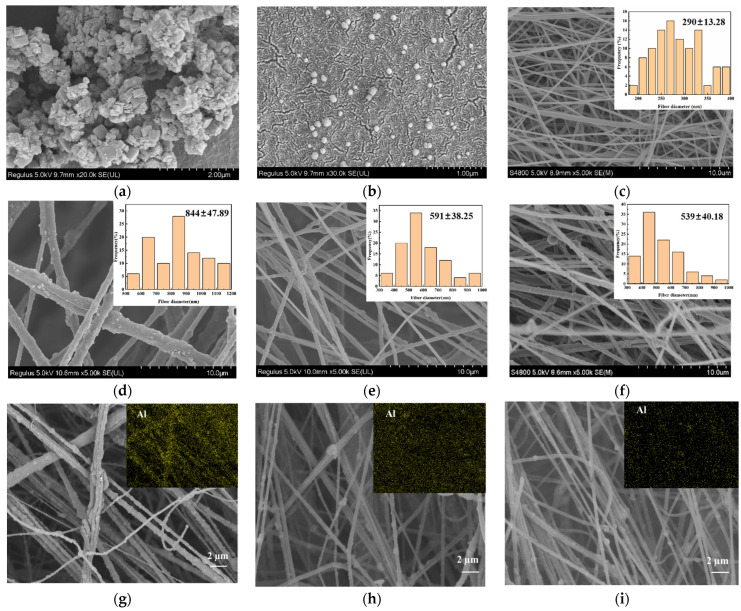
SEM pictures of (**a**,**b**) BM particles, (**c**) PAU, (**d**) BM/PAU-1, (**e**) BM/PAU-2, and (**f**) BM/PAU-3 ENSs and their corresponding nanofiber diameter distributions; EDS of elemental mapping of Al for (**g**) BM/PAU-1, (**h**) BM/PAU-2, and (**i**) BM/PAU-3 ENSs.

The morphologies of BM particles and BM/PAU composite ENSs were observed by using scanning electron microscopy (SEM), and their nanofiber diameter distributions were measured by Image J software (Version 1.54j 12 June 2024). As shown in Figure 1a, the synthesized BM particles were at the nanoscale, and there were agglomerations between the particles due to their high surface energy. Therefore, by dispersing BM particles with ethanol for characterization, it was found that they were circular nanoparticles with a diameter of approximately 35–70 nm (Figure 1b). Figure 1c–f displays that all ENSs exhibited a three-dimensional porous network structure, which could better absorb the electrolytes and further facilitate Li^+^ transport. And their illustrations show the corresponding fiber diameter distributions. It could be seen from Figure 1d–f that the addition of BM particles made the fiber surface of BM/PAU ENSs rough and uneven, and the average nanofiber diameter of these ENSs increased with the increase in BM content. Figure 1g–i shows the energy disperse spectroscopy (EDS) of elemental mapping images of Al in BM/PAU ENSs with different BM contents, where the dispersion level of BM was demonstrated by the signal of Al, indicating that BM particles were uniformly distributed in these ENSs. Moreover, it could be found from Tables S1–S3 that the contents of Al in BM/PAU-X (X=1–3) were 14.155%, 11.069%, and 9.652%, respectively, indicating that the content of Al in ENSs also increased with the increase in BM content.

The average nanofiber diameter of BM/PAU-1 (844 ± 47.89 nm) was higher than that of BM/PAU-2 (591 ± 38.25 nm) and BM/PAU-3 (539 ± 40.18 nm), which was due to the increased viscosity of the spinning solution (Table 1). In addition, with the higher viscosity of the spinning solution, nanofibers were also more likely to adhere during the spinning process, resulting in an increase in the average fiber diameter of ENSs [14]. 

**Figure 2:**
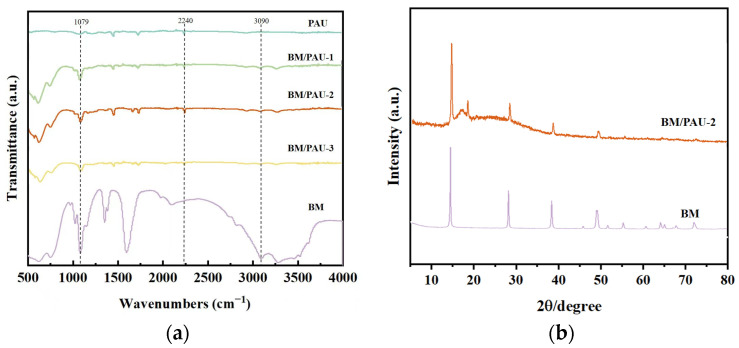
(**a**) FTIR spectra of BM and BM/PAU-X (X=1–3); (**b**) XRD spectra of BM particles and BM/PAU-2.

The chemical structure of BM particles and BM/PAU-X (X=1–3) was verified by using Fourier transform infrared spectroscopy (FTIR). As shown in Figure 2a, all BM/PAU ENSs showed the characteristic infrared absorption peak of C≡N functional group at 2240 cm^−1^, which was the characteristic absorption peak of PAN polymer. Meanwhile, they also showed absorption peaks at 1068 cm^−1^ and 3090 cm^−1^, corresponding to the symmetric bending vibration and asymmetric stretching vibration of O-H in the BM particles [15], respectively. The peak values of these two absorption peaks weakened with the decrease in BM content, indicating that BM particles were successfully loaded onto PAU. Additionally, further characterization of BM particles and BM/PAU-2 was carried out using X-ray diffraction (XRD) analysis, and the results are shown in Figure 2b. It was found that the prepared BM particles exhibited distinct diffraction peaks of the rhombohedral system, with diffraction peaks at 2θ = 14.5, 28.2, 38.4, and 49.2°, corresponding to the (020), (120), (031), and (200) crystal planes of BM, respectively [12]. BM/PAU-2 also exhibited the characteristic peaks of BM crystal phase at 2θ = 14.5, 28.2, 38.4, and 49.2°, indicating that BM particles were well compounded into the PAU ENS, further demonstrating the successful preparation of BM/PAU ENSs. In addition, a broad signal in the range of 2θ = 15°–40° caused by PAU appeared in BM/PAU-2 ENS.

**Figure 3:**
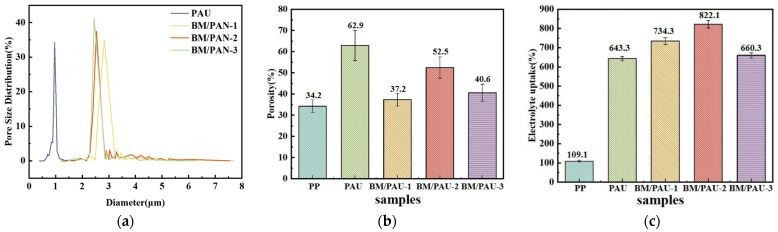
(**a**) Pore size distributions, (**b**) Porosities, and (**c**) Electrolyte uptake rates of different separators.

The pore size distribution of ENSs can affect their porosity, which can be determined by an automatic surface porometer. The fiber diameter of ENSs is an important factor affecting the pore size of ENSs. Generally, with the increase in fiber diameter, the average pore size of ENSs increases [16]. As shown in Figure 3a and Table 2, the average pore sizes of PAU, BM/PAU-1, BM/PAU-2, and BM/PAU-3 were 0.98, 2.90, 2.72, and 2.60 μm, respectively, indicating that the average pore size of all BM/PAU ENSs was larger than that of PAU ENS, due to the fact that their average fiber diameter was larger than that of PAU ENS. Moreover, as the BM content increased, the pore size distribution of BM/PAU ENSs widened. This was because a larger number of nanoparticles would accumulate, resulting in stacked pores with different pore sizes. Meanwhile, with the increase in BM content, the average fiber diameter of BM/PAU ENSs increased (Figure 1d–f), which would lead to an increase in their average pore sizes and a decrease in their pore counts. In general, BM/PAU-2 had more pores and larger pore sizes, which were favorable for the penetration of electrolytes and the transport of Li^+^.

### 2.3. Electrolyte Affinity of ENSs

The porosity of an LIB separator has a remarkable impact on its electrolyte affinity. A higher porosity typically enhances the overall performance of ENSs. As displayed in Figure 3b, compared to PP (34.2%), all ENSs exhibited significantly higher porosity, with porosity of 62.9%, 37.2%, 52.5%, and 40.6% for PAU, BM/PAU-1, BM/PAU-2, and BM/PAU-3, respectively, which was attributed to the interlocking of nanofibers to provide enough space. Among them, the porosities of all BM/PAU ENSs were lower than those of PAU ENS because some pores were partially blocked by BM particles. BM/PAU-2 exhibited the highest porosity among the composite BM/PAU ENSs due to relatively more pores and larger pore sizes (Table 2) [17]. In addition, due to the high viscosity of its spinning solution and excessive BM content, the fiber diameter of BM/PAU-1 was large and its fibers were prone to adhesion during the spinning process, resulting in the minimum porosity. 

**Figure 4:**
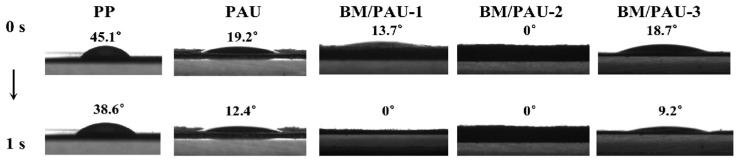
Electrolyte contact angles of different separators.

Electrolyte affinity of separators refers to their adsorption capacity and compatibility to the electrolyte. High electrolyte affinity can provide a higher amount of electrolyte absorption and facilitate the rapid migration of ions in LIBs. The electrolyte affinity of separators is usually evaluated from two aspects: electrolyte wettability and electrolyte uptake rate. Superior electrolyte wettability facilitated the absorption and diffusion of electrolyte inside the separator, providing an effective channel for Li^+^ transport, thereby increasing ionic conductivity and optimizing the electrochemical performances of LIBs. Contact angle measurements were conducted to evaluate the electrolyte wettability of PP and different ENSs. As Figure 4 depicts, the electrolyte wettability of all ENSs was superior to that of PP (45.1°), which was attributed to their porous structure, thus exhibiting good electrolyte affinity. Compared to the PP separator, the lowest contact angle was observed in BM/PAU-2 (0°), which was because its excellent porosity provided more space to store the electrolyte, resulting in its fastest electrolyte uptake rate. Additionally, the OH group of BM had high polarity, which greatly improved the compatibility between the separator and electrolyte [18]. Therefore, the porous structure of BM/PAU-2 offered increased space for electrolyte penetration, while its high electrolyte affinity ensured that the electrolyte could smoothly ingress into these pores.

The electrolyte uptake rate is also an important indicator to judge the electrolyte affinity of a separator, and the higher value means better the affinity between the separator and the liquid electrolyte, which is conducive to the transmission of Li^+^. Figure 3c displayed the electrolyte uptake rates of PP, PAU, and BM/PAU-X (X=1–3) separators, which were 109.1%, 643.3%, 734.3%, 822.1%, and 660.3%, respectively. The higher electrolyte uptake rates of BM/PAU ENSs indicated that ENSs with a three-dimensional network structure were more beneficial for the storage of the electrolyte. Among them, BM/PAU-2 had the highest electrolyte uptake rate, which was consistent with the results obtained from previous measurements of porosity and electrolyte contact angle, confirming that the electrolyte uptake rate of the separator was related to its porosity and electrolyte wettability [19].

### 2.4. Flexibility and Thermal Stability of ENSs

**Figure 5:**
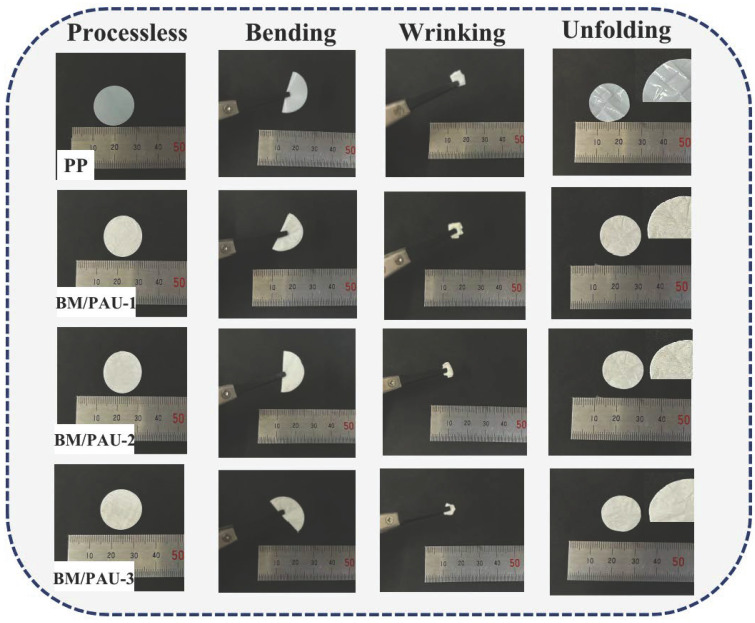
Wrinkle tests of PP and BM/PAU-X (X=1–3) ENSs.

During the assembly, storage and transportation of LIBs, separator deformation caused by various external forces is inevitable [17]. The flexibility of the separator is also a crucial feature that has garnered considerable attention. As shown in Figure 5, after bending and wrinkling tests, a large number of uneven folds were generated on the surface of PP. At the same time, BM/PAU ENSs could return to a relatively flat state after unfolding, and their surfaces were flat with no obvious folds. This wrinkling tests proved that all ENSs had outstanding flexibility, which improved the safety of LIBs during assembly and use.

**Figure 6:**
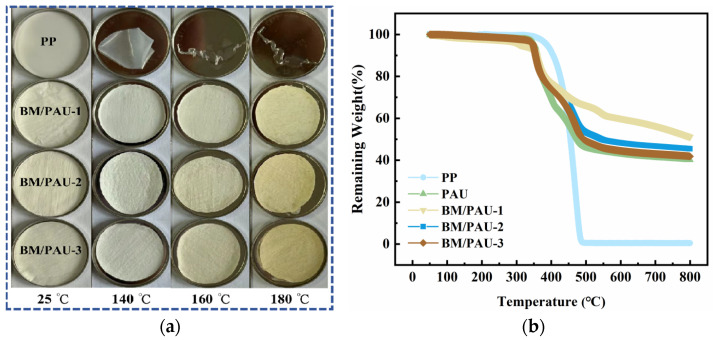
(**a**) Thermal shrinkage of PP and BM/PAU-X (X=1–3) separators after thermal treatment for 30 min at various temperatures; (**b**) TG curves of PP, PAU, and BM/PAU-2 separators.

It is widely known that the dimensional shrinkage of separators can lead to short circuits. LIBs release a lot of heat during charging and discharging, which makes it crucial for separators to maintain dimensional stability under high-temperature conditions. In order to compare the thermal stability of PP and BM/PAU separators, they were placed in an oven at 140 °C, 160 °C, and 180 °C for 30 min, respectively, and then the changes in the dimensions of separators after the thermal treatment were observed and calculated. Figure 6a shows that PP exhibited serious shrinkage (97.89 ± 0.42%) at 140 °C, while it had completely melted at 160 °C and 180 °C. This drastic phenomenon of dimensional thermal shrinkage might lead to short circuits at high temperatures caused by overcharging, causing serious safety problems [20]. In contrast, BM/PAU-X (X=1–3) ENSs basically maintained their original dimensions at 180 °C (6.7 ± 0.3%, 7.2 ± 0.6%, 7.4 ± 0.6%), with only a slight yellowing in color, which was because of the gradual oxidation of -CN in PAN at high temperatures for a long time [21]. Its good thermal stability was mainly due to PAN itself and the introduction of BM particles with good thermal stability. Compared with PP, BM/PAU-X (X=1–3) ENSs had a higher melting point, which ensured their dimensional stability at higher temperatures and avoided their drastic thermal shrinkage, thus enhancing the safety of LIBs. Moreover, due to the excellent thermal stability of BM particles, the higher the BM content, the better the thermal stability of the ENS, resulting in less shrinkage. Accordingly, BM/PAU-1 ENS had the best thermal stability and the smallest thermal shrinkage rate.

The TG curves of PP, PAU, and BM/PAU separators shown in Figure 6b were used to determine their decomposition temperature. It could be seen that PAU and BM/PAU composite ENSs showed a similar downward trend, with a sharp fall in weight loss in the two temperature ranges of 300–350 °C and 350–450 °C, which was due to the oligomerization and cyclization of nitriles in PAN molecules [22,23,24]. When the temperature approached 490 °C, the weight of PP plummeted and its remaining weight approached 0%. In contrast, the remaining weight ratio of PAU, BM/PAU-1, BM/PAU-2, and BM/PAU-3 ENSs were 40.2%, 51.1%, 45.5%, and 41.9%, respectively, further illustrating that BM/PAU ENSs had better thermal stability and the thermal stability of ENS improved with the increase in BM content. Accordingly, BM/PAU-2 had excellent thermal stability and was suitable for the use of a separator in LIBs at higher temperatures.

### 2.5. Electrochemical Performances

Good electrochemical performance is a fundamental requirement for battery separators, which enables them to maintain the stability of structure and performance during operation. It is also an important guarantee for the safe use of separator materials and helps to ensure the overall performance of the battery. One of the most important functions of separators is to provide porous channels for ion transport, and ionic conductivity is an important parameter to characterize this ability. Higher ionic conductivity leads to the improvement of electrochemical reactions and the reduction of internal resistance. 

**Figure 7:**
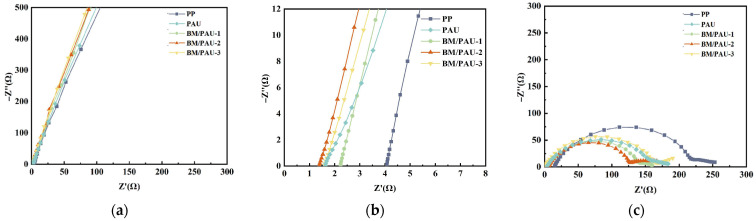
(**a**,**b**) AC impedance spectra of stainless steel symmetrical batteries (SS/separator/SS); (**c**) Nyquist plots of LM symmetric batteries (LM/separator/LM).

Figure 7a,b showed the alternating current (AC) impedance spectra of stainless steel (SS) symmetrical batteries (SS/separator/SS) using PP and BM/PAU-X (X=1–3) separators, respectively, where the intersection of the obtained curve with the actual axis represented the bulk resistance (*R_b_*) of the separator. The *R_b_* values of separators are displayed in Table 3, and their corresponding ionic conductivity (δ) was calculated by EqS4. Compared to PP with a low ionic conductivity of 0.24 mS/cm, BM/PAU-X (X=1–3) exhibited higher ionic conductivity, mainly related to their porous structure and better electrolyte affinity, which was conducive to the rapid migration of Li^+^ between the anode and cathode. In addition, the introduction of an appropriate amount of BM particles also contributed to the improvement of the ionic conductivity. Among BM/PAU ENSs, BM/PAU-2 embodied the smallest *R_b_* and the largest ionic conductivity due to its highest porosity and best electrolyte uptake rate. Therefore, the excellent porous structure and electrolyte affinity endowed BM/PAU-2 with superior ionic transport, which was the key to realizing efficient charging and discharging of LIBs.

Electrochemical impudence spectroscopy (EIS) was used to evaluate the interfacial compatibility between the electrolyte and electrode. Figure 7c displayed the interfacial resistance of symmetrical batteries using lithium metal (LM) as the electrode (LM/separator/LM). The interfacial resistance of LM/PP/LM, LM/PAU/LM, and LM/BM/PAU-2/LM was 222.2 Ω, 176.5 Ω, and 130.7 Ω, respectively. The results showed that BM/PAU-2 ENS exhibited excellent interfacial compatibility because of its best electrolyte affinity, which contributed to the rapid formation of a stable surface between the separator and electrode.

**Figure 8:**
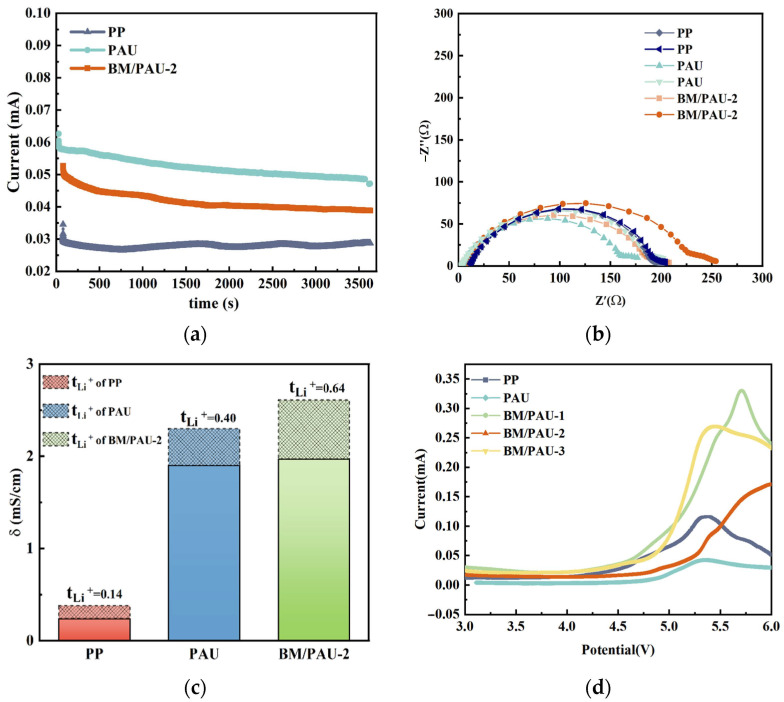
(**a**) Chronoamperometry profiles and (**b**) impedance spectra before and after polarization of LM/separator/LM batteries; (**c**) ionic conductivity and $t_{Li^{+}}$ values of PP, PAU, and BM/PAU-2 separators; (**d**) LSV curves of PP, PAU, and BM/PAU-X (X=1–3) separators at scan rate of 10 mV s^−1^.

Besides the ionic conductivity, the lithium-ion transference number ($t_{Li^{+}}$) is another important indicator on behalf of the lithium-ion transport ability of a separator, which is measured using a conjunction of chronoamperometry and EIS method. Low $t_{Li^{+}}$ values more readily lead to serious concentration polarization and other adverse effects [25]. According to Figure 7c and Figure 8a,b, the $t_{Li^{+}}$ values of PP, PAU, and BM/PAU-2 separators were calculated by EqS5. It was found from Figure 8c that $t_{Li^{+}}$ of BM/PAU-2 (0.64) was greater than that of PAU (0.40) and PP (0.14), which was related to its highest electrolyte uptake rate. In addition, this was attributed to the fact that the H atom in the BM hydroxyl group formed a hydrogen bond with the F atom of ${PF}_{6}^{-}$, which impeded the movement of the ${PF}_{6}^{-}$ [26]. From the comprehensive analysis of ionic conductivity and $t_{Li^{+}}$, the overall electrochemical performance of BM/PAU-2 was better than that of PAU and PP.

**Figure 9:**
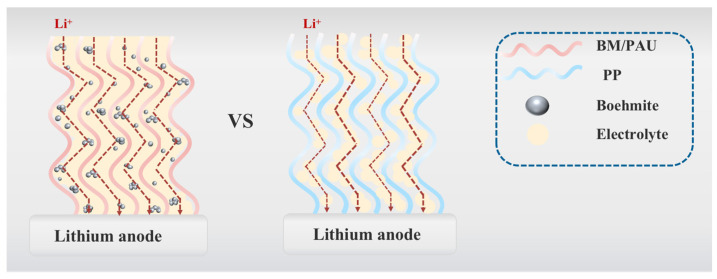
Schematic illustration of lithium-ion transfer in PP and BM/PAU ENSs.

The transport efficiency of Li^+^ is the key to the electrochemical performance of separators. Figure 9 illustrates Li^+^ transport pathways in PP and BM/PAU separators, and explains the importance of electrolyte uptake rate. BM/PAU ENSs with remarkable porosity and electrolyte affinity could store more electrolytes. Therefore, it provided the optimized channels for the transfer of lithium-ions, resulting in outstanding ionic conductivity and $t_{Li^{+}}$.

The linear scanning voltammetry (LSV) curve is of great significance to evaluate the electrochemical stability of a separator in practical application. The wide electrochemical stability window of a separator is beneficial for meeting the long-term stable operation requirements of LIBs. BM/PAU-X (X=1–3) ENSs had wide electrochemical stability windows, which were conducive to meeting the demand for long-term stable operation of LIBs. It could be seen from Figure 8d that the electrochemical stability windows of LIBs assembled with different separators were about 4.4 V (PP), 4.7 V (PAU), 4.6 V (BM/PAU-1), 5.0 V (BM/PAU-2), and 4.6 V (BM/PAU-3), respectively, which suggested that BM/PAU-2 had the most favorable compatibility with the electrolyte and best electrochemical stability in a high voltage condition due to its highest porosity and electrolyte uptake rate, making it more suitable for application in LIBs with high operating voltage. Additionally, BM particles had good compatibility with the electrolyte due to their OH group, further enhancing the electrochemical stability window of BM/PAU-2 and making it have the largest electrochemical stability window. 

### 2.6. Cycling and Rate Performances

**Figure 10:**
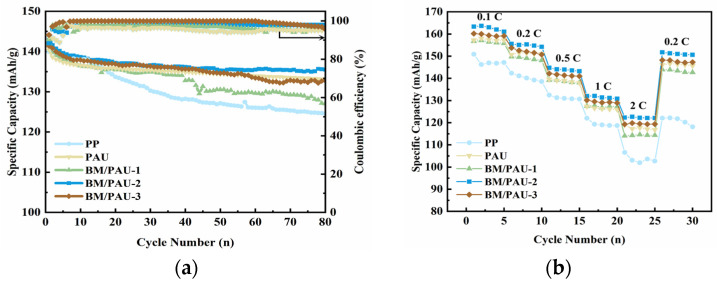
(**a**) Cycling performance at 0.5 C and (**b**) rate performance of LiFePO_4_/separator/LM batteries with different separators.

The cycling performance of batteries (LiFePO_4_/separator/LM) with different separators was measured at the C-rate of 0.5 C, as indicated in Figure 10a. The initial discharge specific capacities of the batteries assembled by BM/PAU-X (X=1–3), PAU, and PP separators were 141.9, 143.6, 140.4, 140.4, and 139.9 mAh/g, respectively. After cycling at 0.5 C for 80 cycles, the specific discharge capacity of the battery assembled by BM/PAU-2 was 135.5 mAh/g, and its capacity retention rate and coulombic efficiency maintained 94.4% and 97.9%, respectively, which were better than those of the batteries assembled by other separators. This was attributed to its highest electrolyte affinity, electrolyte uptake rate, and $t_{Li^{+}}$ of BM/PAU-2, which could be applied to long-term testing.

Figure 10b showed the C-rate performance of LiFePO_4_/separator/LM batteries with different separators at room temperature under a different discharge C-rate. Notably, with the increase in the C-rate, the discharge capacity of the battery gradually decreased. When the C-rate increased to 2 C, the discharge capacity of the battery assembled by BM/PAU-2 was 122.3 mAh/g. But when the C-rate returned to 0.2 C, the specific discharge capacity of the battery assembled with BM/PAU-2 recovered to 151.7 mAh/g, which was higher than that of other separators, exhibiting its outstanding reversibility.

## 3. Materials and Methods

### 3.1. Preparation of BM Particles

Firstly, 6.0 g BM and 60 mL deionized water were put into the inner lining of a para-polystyrene reactor. Then, the assembled hydrothermal reactor was placed in an oven at 180 °C for 16 h. Finally, the synthesized products were washed multiple times with ethanol and dried at 80 °C for 12 h.

### 3.2. Preparation of Spinning Solution

Firstly, a PAU spinning solution with a total mass fraction of 12% using DMF as the solvent, in which the ratio of solute PAN to PU was 8:2 (*w*/*w*), was prepared and stirred for 12 h at room temperature. Afterward, BM particles of different masses were put into the PAU solutions and stirred for 6 h to obtain uniformly dispersed BM/PAU spinning solutions, in which the mass ratios of BM:PAU were 12:15, 6:15, and 4:15, respectively. The viscosity and conductivity of spinning solutions with different BM contents were measured by using a digital viscometer (SNB-1, Ruifang Biotechnology Co., Ltd., Shanghai, China) and a conductivity meter (DDS-307A, INESA Analytical Instrument Co., Ltd., Shanghai, China), respectively.

### 3.3. Batch Fabrication of Nanofiber Separators

**Figure 11:**
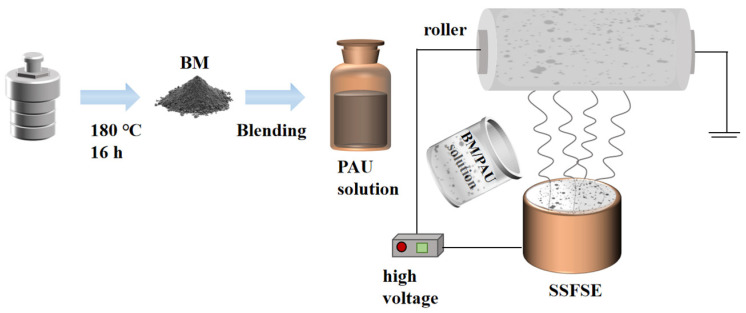
Batch fabrication of BM/PAU ENSs.

As illustrated in Figure 11, BM/PAU ENSs were fabricated in batch by the SSFSE device and used as LIB separators. The SSFSE parameters were set to a spinning voltage of 60 kV, a collection distance of 220 mm, and a drum speed of 340 r/min. And all SSFSE experiments were carried out under suitable temperature (20 °C–30 °C) and relative humidity (50–70%) conditions. The prepared BM/PAU ENSs with a mass ratio of 12:15, 6:15, and 4:15 for BM and PAU were named BM/PAU-1, BM/PAU-2, and BM/PAU-3, respectively.

## 4. Conclusions

In this work, BM/PAU composite ENSs were successfully prepared in batch using SSFSE and applied to LIBs. The effects of BM content on the morphology, structure, and properties of BM/PAU ENSs were discussed. The results illustrated that when the mass ratio of BM and PAU was 6:15, the porosity (52.5%) and electrolyte uptake rate (822.1%) of BM/PAU-2 were the highest, providing effective channels for the transmission of Li^+^, thereby improving the ionic conductivity (1.97 mS/cm) and $t_{Li^{+}}$ (0.64). Meanwhile, BM/PAU-2 had excellent flexibility and thermal stability, which could improve the safety of LIBs during assembly and use. Furthermore, the battery assembled with BM/PAU-2 had the best electrochemical performance, cycling performance, and rate capability, which could be maintained at 135.5 mAh/g after 80 cycles at 0.5 C, with a capacity retention rate of 94.4%, showing its superiority for a long-term cycling test. In summary, the excellent porous structure and electrolyte affinity endowed BM/PAU-2 with outstanding overall performances, which was the key to constructing efficient and safe LIBs.

## Figures and Tables

**Table 1:** Viscosity and conductivity of spinning solutions with different BM contents.

Spinning Solutions of Samples	Mass Ratio of BM and PAU	Spinning Solution Property	
		Viscosity (mPa·s)	Conductivity (µS·cm^−1^)
PAU	/	844 ± 7	678 ± 8
BM/PAU-1	12:15	869 ± 5	649 ± 2
BM/PAU-2	6:15	768 ± 2	550 ± 8
BM/PAU-3	4:15	702 ± 7	526 ± 5

**Table 2:** Pore size distributions of different ENSs.

Separators	PAU	BM/PAU-1	BM/PAU-2	BM/PAU-3
Pore size (µm)	0.79–1.04	2.61–3.71	2.45–3.41	2.38–3.18
Average pore size (µm)	0.98	2.90	2.72	2.60
Pore count (/cm^2^)	2373	1068	1580	1864

**Table 3:** *R_b_* and δ for PP and BM/PAU-X (X=1–3) assembled batteries.

Separators	PP	PAU	BM/PAU-1	BM/PAU-2	BM/PAU-3
Thickness (mm)	0.025 ± 0.002	0.083 ± 0.007	0.068 ± 0.005	0.070 ± 0.008	0.074 ± 0.010
*R_b_* (Ω)	4.00	1.70	2.24	1.39	1.61
δ (mS/cm)	0.24	1.90	1.19	1.97	1.79

## Data Availability

The data presented in this study are available on request from the corresponding author.

## Supplementary Materials

The following supporting information can be downloaded at: https://www.mdpi.com/article/10.3390/molecules29163938/s1, Table S1: EDS analysis of BM/PAU-1; Table S2: EDS analysis of BM/PAU-2; Table S3: EDS analysis of BM/PAU-3.
