# Supplementary material for: Batch Preparation and Performance Study of Boehmite-Based Electrospun Nanofiber Separators for Lithium-Ion Batteries
Source: Molecules. 2024 Aug 21;29(16):3938. doi: 10.3390/molecules29163938 (PMC11356863; doi:10.3390/molecules29163938)
Supplement: Supplementary file 1 [file molecules-29-03938-s001.zip › molecules-3152135-supplementary.pdf]

Batch Preparation and Performance Study of Boehmite-based Electrospun Nanofiber Separators for Lithium-ion Batteries

Supporting Information

Wenfei Ding [1†], Yuxing Liu [1†], Lan Xu[1,2*]

[1]National Engineering Laboratory for Modern Silk, College of Textile and Engineering, Soochow University, 199 Ren-ai Road, Suzhou 215123, China

[2]Jiangsu Engineering Research Center of Textile Dyeing and Printing for Energy Conservation, Discharge Reduction and Cleaner Production (ERC), Soochow University, Suzhou 215123, China

* Corresponding authors: lanxu@suda.edu.cn (L.X.).

† First Author: Wenfei Ding and Yuxing Liu contributed equally to this paper.

Raw Materials

Boehmite (BM) was supplied by Ho-jun polymer material Co., Ltd (China). Polyacrylonitrile (PAN, MW=150000) was supplied by Beijing Lark Branch Co., Ltd (China). N,N-dimethylformamide (DMF) (Analytical Reagent) was purchased from Shanghai Chemical Reagent Co., Ltd (China). Polyurethane (PU) was supplied by Yuyao Bangchao new materials Co., Ltd (China). The commercialized polypropylene (PP) separator (Celgard 2400) was used for comparison, which was provided by Suzhou Shengernuo Technology Co., Ltd (China). The electrolyte, 1 M solution of lithium hexafluorophosphate ($LiPF_6$) in ethylene carbonate, dimethyl carbonate and ethylmethyl carbonate (EC+DMC+EMC, 1:1:1 by volume), was supplied by Suzhou Dodo Chemical Technology Co., Ltd. Poly(vinylidene fluoride) (PVDF), carbon black (Super P-C45), $LiFePO_4$, and N-methyl-2-pyrrolidone (NMP) were obtained from Ferro Corp.

* Corresponding author. Email address: lanxu@suda.edu.cn (L. Xu)

Battery Assembly

Lithium iron phosphate ($LiFePO_4$) cathode preparation process: Firstly, PVDF and carbon black were added into N-methyl pyrrolidone (NMP) solvent in sequence, and after the previous two were fully mixed and stirred for 2 h, then $LiFePO_4$ was added and stirred for 8-10 h. Subsequently, the homogeneously mixed slurry was coated on the aluminium foil of the collector, and then the cathode was dried for 12 h, with the temperature set at 110 °C to remove the excess solvent and fix the material structure. 110 °C to remove excess solvent and fix the material structure. Finally, the cathode with flat surface were obtained and set aside with 2 mg of active material.

The battery shells were ultrasonically cleaned twice with ethanol and dried at 60 °C, and then placed in a glove box filled with argon gas (both the O_2 and H_2O content were less than 0.01 ppm). All composite BM/PAU ENSs and $LiFePO_4$ cathode were dried for half an hour to remove water before assembly.

Characterization of Nanofiber Separators

The morphology and structure of samples were characterized by scanning electron microscopy (SEM, Regulus8100). 5 SEM images and 20 nanofibers at random in each SEM image were chosen for nanofiber diameter distribution analysis through ImageJ software (v 1.54 h). Energy Disperse Spectroscopy (EDS) elemental mapping images were performed on Table SEM (TM3030, JPN).

Fourier transform infrared spectroscopy (FTIR) spectra of samples were performed on a Nicolet5700 spectrometer (Perkin Elmer Spectrum GX, USA) at the range of 400-4000cm^{-1}. The crystalline structure of samples was elucidated using X-ray diffraction (XRD, D8 Advance, German) analysis with a diffraction angle (2θ) in the range of 5°-80°. The thermostability of samples was measured by thermogravimetric analysis (TGA) from room temperature to 800 °C at a heating rate of 20 °C/min in N_2 atmosphere (TG/DTA, TG/DTA5700, USA). The pore size distribution of ENSs were measured through Capillary Flow Porometer (CFP-1500a).

To determine the thermal shrinkage rate of nanofiber separators, the separators with a diameter of 19 mm were heated in a constant temperature drying oven for 30 min at

each temperature point, where these set temperature points were 140 °C, 160 °C and 180 °C, respectively. Then took out the separators, measured their sizes before and after heating with a ruler, and calculated the thermal shrinkage rate (*S*) of separators according to the following formula (1):

$$S = \frac{D_1 - D_2}{D_1} \times 100\% \tag{1}$$

where D_1 is the separator diameter at room temperature (19 mm), D_2 is the minimum diameter of the separator after heat treatment for 30min.

The porosity (*P*) of separators was tested by soaking in n-butanol, and calculated according to the following formula (2):

$$P = \frac{m_T - m_s}{\rho_1 \times \pi R^2 \times h} \times 100\% \tag{2}$$

where m_T is the mass of separator after absorbing n-butanol, m_S is the dry mass of separator, ρ_1 is the density of n-butanol (0.81g/cm^3), R is the radius of separator (9.5 mm), h is the thickness of separator.

The electrolyte affinity of separators could be characterized by measuring the contact angle between separator and electrolyte, which was determined using a contact angle meter (Krüss DSA 100, German Cruz company, German), and the volume of each drop of electrolyte was set to 5 μL. After each sample was tested at different positions for 5 times, the angle average value was obtained. In addition, the electrolyte adsorption rate (U_{ptake}) of separators with a diameter of 19 mm was tested by soaking in electrolyte for 2 h, and calculated according to the following formula (3):

$$U_{ptake} = \frac{W_w - W_d}{W_d} \times 100\% \tag{3}$$

where Ww is the weight of separator after absorbing the electrolyte, Wd is the dry weight of the separator.

The pore size distribution of separators was determined by an automatic surface porometer. Before the test, the dried separator should be cut into a circular sample film with a diameter of about 25 mm on the measuring table, cut off the excess part, and then infiltrate the sample to be tested with drugs, and put it into the instrument to determine the hole structure and aperture size.

Eletrochemical Tests

Ionic conductivity can characterize the ability of separators to allow lithium-ions

(Li^+) to pass through. For measuring the ionic conductivity of separators, a separator was placed between two stainless steel (SS) electrodes and assembled into a coin battery (CR2025) in the glove box filled with argon. Then the electrochemical workstation (SP-300, Bio-Logic company, France) was applied to test the battery (SS|separator|SS battery) by using the alternating current (AC) impedance method. AC impedance spectra of batteries were determined with the test frequency range from 1 to 10^6 Hz at an amplitude of 5 mV. And the ionic conductivity of separators (δ) could be calculated using the following equation (4):

$$\delta = \frac{L}{R_b \times A} \tag{4}$$

where L is the thickness of separator (cm), R_b represents the bulk resistance of separator confirmed from the Nyquist plot of AC impedance spectrum (Ω), and A is the area of SS electrode ($1.6 \times 1.6\ \mathrm{cm}^2$), respectively.

For measuring the lithium ion transference number of separators, a coin battery with lithium metal (LM) as electrodes (LM|separator|LM battery) was assembly according to the above method, and then it was tested by the electrochemical workstation (SP-300) with a polarization voltage (ΔV) of 10 mV. The lithium-ion transference number (t_{Li^+}) was calculated using the following equation:

$$t_{Li^+} = \frac{I_S R_b^0 [\Delta V - I_0 R_1^0]}{I_0 R_b^s [\Delta V - I_s R_1^s]} \tag{5}$$

where I_0 and I_s are the initial and steady state currents measured by the chronoamperometry, respectively, R_b^0 and R_b^s are the bulk resistances before and after the potentiostatic polarization measurement, and R_1^0 and R_1^s are the interfacial resistances before and after the potentiostatic polarization measurement.

The electrochemical stability of separators was characterized by using linear scanning voltammetry (LSV). Based on the coin battery assembled with SS as the working electrode and LM as the counter electrode (SS|separator|LM battery), LSV tests were carried out using the electrochemical workstation (SP-300) at a scanning rate of 1mV/s within the range of 0-6 V at room temperature.

The charge-discharge cycle performance of $LiFePO_4$|separator|LM batteries was conducted by a battery testing system (CT-4008T-5V10Ma-164, Shenzhen Xinwei Electronics Co., Ltd, China) with a potential window of 2.5-4.0 V at a current density of 0.5 C.

In order to evaluate the rate capability of $LiFePO_4$|separator|LM battery, the battery was charged to 4.0 V and discharged to 2.5 V galvanostatically from 0.1 C, 0.2 C, 0.5 C to 2 C (n C refers to the applied current when the fully charged battery is totally discharged within 1/n h) at room temperature.

Table S1 EDS analysis of BM/PAU-1.

Element	Weight %	Weight % σ	Atomic %
**Carbon**	53.455	0.495	71.538
**Oxygen**	18.823	0.448	18.911
**Aluminum**	14.155	0.180	8.433
**Platinum**	13.567	0.322	1.118

Table S2 EDS analysis of BM/PAU-2.

Element	Weight %	Weight % σ	Atomic %
**Carbon**	68.157	0.931	85.760
**Oxygen**	7.416	0.750	7.005
**Aluminum**	11.069	0.313	6.200
**Platinum**	13.358	0.701	1.035

Table S3 EDS analysis of BM/PAU-3.

Element	Weight %	Weight % σ	Atomic %
**Carbon**	66.238	1.032	85.672
**Oxygen**	7.686	0.826	7.463
**Aluminum**	9.652	0.316	5.557
**Platinum**	16.424	0.812	1.308
